# The effects of binge-pattern alcohol consumption on orthodontic tooth movement

**DOI:** 10.1590/2176-9451.19.6.093-098.oar

**Published:** 2014

**Authors:** Cristiano Miranda de Araujo, Aline Cristina Batista Rodrigues Johann, Elisa Souza Camargo, Orlando Motohiro Tanaka

**Affiliations:** 1 Catholic University of Paraná, PhD resident in Dentistry, Catholic University of Paraná (PUC-PR); 2 PUC-PR, School of Dentistry, Associate professor, School of Dentistry, PUC-PR; 3 PUC-PR, School of Dentistry, Full professor, School of Dentistry, PUC-PR

**Keywords:** Tooth movement, Orthodontics, Bone remodeling

## Abstract

**OBJECTIVE:**

This study aimed to assess tissue changes during orthodontic movement after binge-pattern ethanol 20% exposure.

**METHODS:**

Male Wistar rats (n = 54) were divided into two groups. The control group (CG) received 0.9% saline solution, while the experimental group (EG) received 20% ethanol in 0.9% saline solution (3 g/kg/day). On the 30^th^ day, a force of 25 cN was applied with a nickel-titanium closed coil spring to move the maxillary right first molar mesially. The groups were further divided into three subgroups (2, 14 and 28 days). Tartrate-resistant acid phosphatase and picrosirius were used to assess bone resorption and neoformation, respectively. Data were compared by two-way ANOVA, Tukey's HSD, Games-Howell and chi-square test. Significance level was set at 5%.

**RESULTS:**

There was a decrease in the number of osteoclasts in EG at day 28. The percentage of collagen showed no interaction between group and time.

**CONCLUSION:**

Binge-pattern 20% ethanol promoted less bone resorption at the end of tooth movement, thereby suggesting delay in tooth movement.

## Abstract (pt)

**OBJETIVO:**

objetivou-se avaliar as alterações teciduais decorrentes da administração de etanol a 20% no padrão binge, durante o movimento ortodôntico.

**MÉTODOS:**

foram utilizados ratos Wistar machos (n = 54), divididos em dois grupos, sendo Grupo Controle (GC), com administração de soro fisiológico a 0,9%; e Grupo e Experimental (GE), com administração de etanol a 20% em soro fisiológico a 0,9%, no volume de 3g/kg/dia. Após o 30º dia de administração, foi aplicada força de 25cN com mola fechada de níquel-titânio para mover o primeiro molar superior direito para mesial. Os grupos foram subdivididos nos subgrupos 2, 14 e 28 dias, correspondendo ao número de dias de movimentação dentária. Utilizou-se as colorações de fosfatase ácida-tartarato resistente e picrosírius para avaliar reabsorção óssea e neoformação óssea, respectivamente. Os dados foram comparados por ANOVA a dois critérios, Tukey HSD e Games-Howell, ao nível de significância de 5%.

**RESULTADOS:**

verificou-se diminuição no número de osteoclastos no GE II no 28º dia. A percentagem de colágeno não demonstrou alteração na interação grupo x tempo.

**CONCLUSÕES:**

o etanol no padrão binge a 20% promoveu menor reabsorção óssea no final da movimentação dentária, sugerindo atraso na movimentação dentária.

## INTRODUCTION

Alcohol abuse affects approximately 14 million North Americans.^1^ Ethanol is the main component of alcoholic beverages, and it is considered to be toxic not only to vital organs, but also to hard tissues, such as bones. Chronic alcohol consumption is associated with pathological effects on bone and tissue integrity, which complicates post-injury or surgery repair processes in addition to acceleration of osteoblast apoptosis.^2^^,^ 3

Binge-pattern alcohol consumption in humans is characterized by excessive consumption within a short period of time, with approximately five or more drinks on a single occasion for men and four for women.^4^^,^ 5 According to Callaci et al,^6^ experimental binge-pattern ethanol consumption can be mimicked by administering ethanol injections four days a week, followed by three days during which no alcohol is administered. Intraperitoneal (IP) injections are well tolerated by rats and cause minimal stress. Another advantage of this route is that it achieves a high concentration of alcohol in blood and in a controlled environment. Additionally, it has minimal effects on rat's body weight.

Callaci et al^7^ administered 20% binge-pattern ethanol in rats and found decreased mineral density in the vertebrae, both in cortical and cancellous regions, as well as decreased compressive strength. Similarly, they reported that treatment with 20% ethanol had varying effects on different bone regions, i.e., lumbar vertebrae proved more resistant than the tibia. Callaci et al^6^ also observed that, from the third week of binge-pattern 20% ethanol exposure on, bone mineral density of the femur and lumbar spine decreased significantly.

Orthodontic tooth movement (OTM) is characterized by sequential reactions to biomechanical forces that induce changes in periodontal tissue and are related to bone remodeling by activation of alveolar bone resorption on the pressure side and consequent bone apposition on the traction side.^9^^,^ 10 ^,^ 11 Ethanol-induced imbalance between the processes of bone formation and resorption directly affect bone repair.^15^ To date, there have been no reports in the literature regarding the influence of ethanol on OTM.

Therefore, the objective of our study was to assess the tissue changes occurring during OTM in the periodontal ligament and alveolar bone adjacent to the mesial and distal areas of maxillary right first molar after administration of 20% ethanol. We particularly assessed bone resorption and neoformation.

## MATERIAL AND METHODS

This project was approved by PUC-PR Ethics Committee on Animal Use. A total of 54 male, 9-week-old Wistar rats (*Rattus norvegicus albinus*), weighting approximately 300-350 g, was used. Temperature remained between 19 °C and 22 °C with a 12/12-hour light/dark photoperiod. The rats were provided with crushed food and water *ad libitum*. To observe changes in weight, the animals were weighed weekly with the aid of an electronic precision scale (Gehaka - BG 4001, São Paulo, Brazil).

The animals were randomly divided into two groups (27 rats per group): The control group (CG) received 0.9% saline solution in a volume similar to that given to the experimental group, whereas the experimental group (EG) received 20% ethanol (w/v) in 0.9% saline solution (3 g/kg/day).^6^ These groups were further subdivided into three subgroups (2, 14 and 28 days; n = 9/group),which corresponded to the day of animal death after applying orthodontic force, so as to characterize the evolution of OTM over time.

Administration of solutions began 30 days before the orthodontic appliance was installed and continued until animal's sacrifice. It was performed intraperitoneally and designed so as to mimic binge drinking. Ethanol was administered four days a week, followed by three days of abstinence.^6^

The device used to induce OTM consisted of a nickel-titanium closed coil spring (G&H^(r)^ Wire - Franklin, Indiana, USA) attached to maxillary right first molar and central incisors of all animals, which produced a 25-cN reciprocal force.^14^ Measurement of the force produced by the coil spring was standardized by means of a calibrated dynamometer (Haag-Streit AG, Switzerland Koeniz, Switzerland). After initial activation, the coil spring was not reactivated during the experimental period; however, its position was checked on a daily basis.

The animals were sacrificed with an intraperitoneal overdose of anesthetic (5.4 ml/kg ketamine). Then, the mandible of each animal was removed, dissected and sectioned at the midline. Right hemimaxilla remained in 10% formaldehyde solution for 24 hours for proper fixation. After two months of demineralization, animals' maxilla was further fixed in 4.13% ethylenediamine tetraacetic acid solution (Biotec Analytical Reagents, Pinhais, Brazil), processed and embedded in paraffin. A total of 15 cross-sections were cut on the cervical third of the mesiobuccal root of maxillary first molars with a microtome at 4 µm, the occlusal surface of the molar parallel to the microtome and 60-µm intervals between sections.

The slides were stained with picrosirius and tartrate-resistant acid phosphatase (TRAP). Five sections were used for each technique.

Picrosirius staining was performed as follows: After deparaffinization in xylene, the sections were hydrated in ethanol and incubated for 1 hour in a solution of Sirius Red (Direct Red 80, diluted to 0.19% in saturated picric acid, Aldrich Chemical Company, Milwaukee, USA) at room temperature, followed by washing with distilled water, counterstaining with Harris hematoxylin, dehydrating in increasing alcohol solutions, deparaffinizing in xylene and mounting in Entellan.

For the TRAP technique, we used the TRAP Sigma 387A kit (Sigma-Aldrich Chemicals, St. Louis, Missouri, USA), following the manufacturer's recommendations.

Picrosirius-treated histological slides were assessed under light microscopy. Images were obtained using an Olympus BX-50 microscope (Olympus, Tokyo, Japan) equipped with Olympus U-Pot^(r)^ polarized lens (Olympus, Tokyo, Japan) coupled to a Dino-Lite^(r)^ microcamera (AmMo Electronics Corporation, New Taipei City, Taiwan) at a magnification of 100x. Images were analyzed with the Image Pro Plus morphometry program version 4.5 (Media Cybernetics, Rockville, Maryland, USA) to determine the percentage of areas of immature and mature collagen.^13^ Type I collagen (mature) appeared red-orange, while type III collagen (immature) was yellowish-green.^15^ The bone adjacent to the distal surface of the root was chosen for evaluation, as, during OTM, bone is deposited in the alveolar wall on the traction side.^13^

The TRAP-stained sections were used to identify osteoclasts and to determine bone resorption quantitatively. Thus, TRAP-positive multinucleated cells in the periodontal ligament adjacent to the alveolar bone were considered as functional osteoclasts. These cells were quantified^16^ by means of obtaining five images of the mesial region of the root, totaling an area of 942,813.00 µm² of the periodontal ligament. An Olympus BX-50 microscope (Olympus, Tokyo, Japan) coupled to a Dino-Lite^(r)^ microcamera at 400 x magnification.^17^ Images were analyzed with Image Pro Plus software, version 4.5 (Media Cybernetics, Silver Spring, Maryland, USA), using a counting grid. We calculated the mean of the five sections to obtain the average number of osteoclasts.

Reproducibility power was analyzed. Dahlberg error was less than 1.8%, thereby indicating that the estimate of random error was reliable.

Statistical analysis was performed using SPSS software (version 16.0, SPSS IBM, Armonk, New York, USA). Significance level for all tests was set at 0.05.

To compare the mean values of dependent variables, in other words, the percentage of type I collagen in bone tissue and the number of osteoclasts according to the interaction between group and time, we initially tested the data for normal distribution and homogeneity of variances among the different treatments. To this end, Shapiro-Wilk test and Levene's test were used.

Since groups showed normal distribution (P > 0.05), mean values were compared by means of two-way ANOVA (group and time) with full factorial design. When ANOVA revealed differences and when treatment presented homogeneity of variance, we performed Tukey's HSD test for multiple comparison. For heterogeneous variance, we employed Games-Howell multiple comparison tests.

## RESULTS

## Bone resorption

**Figure 1.**
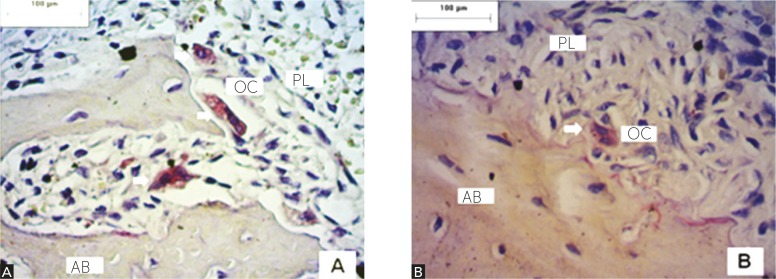
Photomicrographs of histological slides in CG (A) and EG (B) on the 28th day after orthodontic appliance installation. Fewer osteoclasts were observed in the EG on the side where pressure was applied to the periodontal ligament of the mesiobuccal root of the right maxillary first molar. AB: alveolar bone; PL: periodontal ligament; OC: osteoclasts. White arrows indicate A B TRAP-positive cells (TRAP, magnification 400x).

The interaction between group and time revealed statistically significant difference (P < 0.05). EG showed a smaller number of osteoclasts than CG when they were compared on day 28 (Table 1, Fig 1).

**Table 1.** Variables mean and standard deviation: Number of osteoclasts, percentage of type I collagen and weight variation in control (CG) and experimental (EG) groups.

Groups/Variables	Mean ± SD		Comparison
	CG	EG	CG x EG
Number of osteoclasts			
2 days	1.7375 ± 2.05492	2.6286 ± 1.17716	0.971
14 days	4.7250 ± 3.24643	3.8571 ± 2.36492	0.999
28 days	7.0000 ± 3.92641	2.1571 ± 1.72516	0.012*
Percentage of type I collagen			
2 days	86.1425 ± 8.48060	66.1814 ± 15.9878	0.179
14 days	78.5175 ± 17.6788	70.1642 ± 18.7859	0.968
28 days	85.7328 ± 9.10578	75.8685 ± 15.2132	0.932
Weight variation			
2 days	15.7863 ± 4.25056	6.0014 ± 5.31286	0.005*
14 days	10.5050 ± 22.8312	14.1914 ± 4.39931	0.852
28 days	14.2486 ± 2.22475	6.1529 ± 5.77777	0.055

* P < 0.05.

## Bone neoformation

**Figure 2.**
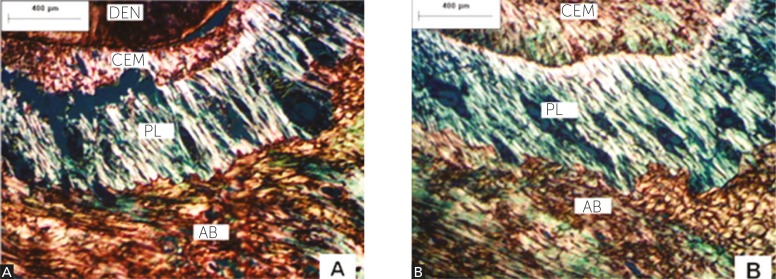
Photomicrographs of histological slides on the distal surface of mesiobuccal root of right maxillary first molar in control (A) and experimental (B) groups on the 28th day after orthodontic appliance installation. There were no statistically significant differences in the group-time interaction. DEN: dentin; CEM: cementum; PL: periodontal ligament; AB: alveolar bone (picrosirius, magnification 100x).

When the percentage of type I collagen was assessed, no statistically significant difference (P > 0.05) was observed based on group-time interaction (Table 1, Fig 2).

## Weight

We found statistically significant weight difference between EG and CG on day 2 (P < 0.05) (Table 1).

## DISCUSSION

Alcohol consumption during adolescence and young adulthood is considered an important public health issue in the United States.^18^^,^ 19 However, despite evidence showing that a significant number of adolescents and young people tend to binge drink ethanol, most studies about the effects of ethanol action on bone metabolism have used chronic consumption models.^8^ Based on these data, we decided to investigate binge pattern which is a more common pattern of alcohol consumption among teenagers and college students,^19^ an age group which often undergo orthodontic treatment.

The methods described in the literature have employed varying concentrations of ethanol and different application times to assess the effects of ethanol on bone tissue and neoformation. Studies on the effects of ethanol on bone tissue have used concentrations ranging from 5% to 20% for periods of 4 to 12 weeks.^2^^,^ 7 ^,^ 8 ^,^ 20 ^,^ 21 No reports associating the effects of ethanol and OTM were found; thus, we used 20% ethanol of which effects on bone neoformation are widely known.^2^^,^ 6 ^,^ 7 ^,^ 8 ^,^ 22

OTM is predominantly mediated by the periodontal ligament. For this reason, periodontal health is essential for OTM to occur without causing deleterious effects to the patient. Dantas et al^23^ stated that ethanol consumption is a risk factor for periodontal health as it promotes local inflammation in gingival tissues. Nevertheless, Liberman et al^24^ reported a dose-dependent relationship between bone loss and ethanol consumption. They also found that low concentrations of ethanol do not significantly lead to alveolar bone loss. Conversely, high concentrations may aggravate bones loss, even in the absence of stainless steel ligature ties which may induce periodontal disease. Accordingly, Souza et al^25^ and Porto et al^26^ also detected the harmful potential of ethanol in periodontal bone tissues. 

In the present study, we observed that on the 28^th^ day after the orthodontic appliance was installed, there was a decrease in the number of osteoclasts in the EG group (P < 0.05) compared to the CG group. There have been reports that ethanol promotes increased resorptive activity; however, the maximal time of application in these studies was four weeks.^6^^,^ 7 ^,^ 8 Preedy et al^27^ assessed the influence of ethanol applied for more than four weeks, and found a decrease in urinary DPD excretion after six weeks of consumption. Accordingly, we observed a statistically significant decrease in the number of osteoclasts at day 28, after six weeks of ethanol exposure. These changes suggest that OTM could be delayed by decreased bone resorption and that ethanol could influence osteoclast activity over time.

Approximately 90% of organic bone matrix consists of type I collagen degraded during bone resorption and replaced by immature fibers composed of type III collagen.^16^^,^ 24 Callaci et al^6^ assessed the effects of ethanol on bone metabolism and found an increase in type I collagen degradation and a corresponding decrease in bone mineral density. Conversely, Maran et al^25^ found that there was no reduction in type I collagen. Similarly, we did not find differences in the percentage of type I collagen in alveolar bone (P ≥ 0.05). These results suggest that ethanol does not influence the processes of collagen deposition and bone neoformation.

We observed statistically significant differences in weight (P < 0.05) at day 2. EG II group showed greater weight variation than CG. Lauing et al^8^ reported that factors such as animal health after intraperitoneal injection, reduced food intake of animals exposed to ethanol and the direct effect of ethanol on the ability of rats to transform dietary nutrients into body weight might have directly influenced the difference in weight gain between control and experimental groups.

The effects of ethanol on bone remodeling remain controversial, but the common hypothesis is that ethanol affects bone metabolism. Differences in variables such as age and time of ethanol consumption could explain discrepant results. In addition, no consensus has yet been reached on which factor, whether increased resorption or decreased neoformation, acts as the major mediator inducing bone loss as a result of ethanol consumption.^12^ Nevertheless, we found that ethanol promoted an imbalance in bone resorption. Additionally, its effects must be thoroughly considered from an orthodontic viewpoint, since tooth movement is a bone-dependent process.

Further studies should be performed in order to find out how ethanol affects bone remodeling. In the present study, we showed that 20% ethanol influences bone metabolism due to decreasing the number of osteoclasts when an orthodontic force is applied. Caution should be taken when applying orthodontic force in individuals who binge drink ethanol, as this substance can delay bone remodeling processes and possibly increase orthodontic treatment total time.

## CONCLUSION

Ethanol does not influence the processes of collagen deposition or bone neoformation.

Binge-pattern 20% ethanol consumption promotes a decrease in resorption at the end of OTM.

Ethanol affects bone metabolism, thereby suggesting delay in OTM.
